# Tiantian capsule alleviates low-fiber diet-induced constipation with improved 5-HT-associated gastrointestinal motility and gut microbiota remodeling

**DOI:** 10.3389/fphar.2026.1755455

**Published:** 2026-08-27

**Authors:** Tingyu Zhang, Jing Sun, Tiantian Wang, Jian Zhang, Qingyuan Liu, Chayanis Sutcharitchan, Lili Li, Boyang Wang, Yinqing Li, Zhiqi Yin

**Affiliations:** 1 Bioinformatics Division, Department of Automation, Tsinghua University, Beijing, China; 2 Department of TCMs Pharmaceuticals, School of Traditional Chinese Pharmacy, China Pharmaceutical University, Nanjing, Jiangsu, China; 3 Affiliated Hospital of Integrated Traditional Chinese and Western Medicine, Nanjing University of Chinese Medicine, Nanjing, Jiangsu, China; 4 Hebei Technology Innovation Center of Precise Nutrition and Health, Hebei Yuzhilin Pharmaceutical Co., Ltd., Shijiazhuang, Hebei, China

**Keywords:** 5-HT, constipation, gut microbiota, low-fiber diet, tiantian capsules

## Abstract

**Background:**

Insufficient dietary fiber intake is closely associated with functional constipation. Although laxatives are widely used, their long-term or inappropriate use may cause adverse effects such as diarrhea and electrolyte imbalance. Tiantian Capsule (TTC) is a marketed health food product with reported laxative effects. However, its effects on low-fiber diet (LFD)-induced constipation and the associated biological changes remain incompletely understood.

**Purpose:**

This study aimed to evaluate the effects of TTC on LFD-induced constipation and to investigate its experimentally supported mechanisms.

**Methods:**

An LFD-induced constipation rat model was established and evaluated using fecal parameters, gastrointestinal (GI) transit assessment, and intestinal histological staining. Colonic transcriptomic profiling was performed to identify potential biological processes associated with TTC intervention. Fecal microbiota profiling and enzyme-linked immunosorbent assay (ELISA) were further used to assess microbiota changes and motility-related GI hormones/neurotransmitters.

**Results:**

TTC improved defecation function and restored intestinal histopathological changes in LFD-induced constipation. Colonic transcriptomic analysis suggested that TTC intervention was associated with changes in serotonergic signaling and intestinal immune-related pathways. Consistently, TTC improved GI transit, modulated serum 5-HT and other GI hormones/neurotransmitters levels, and restored fecal microbiota diversity and composition.

**Conclusion:**

This study suggests that TTC improves LFD-induced constipation-like phenotypes, accompanied by changes in 5-HT-associated GI motility regulation and fecal microbiota composition, warranting further exploration and development.

## Introduction

Constipation is a common clinical condition characterized by reduced bowel movement frequency, hard stools, excessive straining during defecation, a sensation of anorectal obstruction, the need for manual evacuation, and a feeling of incomplete evacuation. It affects a substantial proportion of the global population (Pare et al., 2001; Markland et al., 2013). Although constipation is usually not life-threatening, it can markedly impair quality of life and lead to increased healthcare costs (Bharucha and Wald, 2019). Current management strategies mainly include lifestyle modification, dietary fiber supplementation, and pharmacological therapies such as laxatives, while surgical intervention is reserved for selected patients with severe refractory constipation. However, existing treatments may be limited by inadequate efficacy, poor long-term adherence, or adverse effects such as diarrhea, abdominal discomfort, and electrolyte imbalance (Camilleri and Brandler, 2020; Inoue et al., 2017). Therefore, safe and effective therapeutic options for constipation remain needed.

Tiantian Capsule (TTC) is a marketed health food product formulated from several plants and fungi used in medicines and foods, including *Aloe vera* (AV, Luhui), *Wolfiporia cocos* (PC, Fuling; syn. *Poria cocos*), *Cucurbita moschata* (CM, Nangua) and *Tremella fuciformis* (TF, Yiner), together with maize-derived xylooligosaccharides. AV has been widely used in functional foods, pharmaceuticals, and cosmetics, and has been shown to possess laxative, antioxidant, and anti-inflammatory activities (Yu et al., 2024). PC and TF were documented to regulate the spleen and stomach in the Compendium of Materia Medica, an ancient Chinese pharmacological classic. CM is rich in dietary fiber and micronutrients, and studies have shown its potential medicinal value, including anti-obesity and anti-diabetes effects (Men et al., 2021). Previous studies have reported that TTC alleviates loperamide-induced constipation (Li et al., 2021). Constipation can arise from various causes, with insufficient dietary fiber intake being a primary contributor (Gill et al., 2021). Low-fiber diet (LFD)-associated constipation is also accompanied by alterations in gut microbiota composition compared with healthy conditions (Ceresola et al., 2018). Therefore, whether TTC improves LFD-induced constipation and the underlying mechanisms remain unclear.

Constipation involves impaired gastrointestinal (GI) motility, altered mucosal function, and gut microbiota disturbance. Because TTC is a complex commercial product containing botanical/fungal drugs and maize-derived xylooligosaccharides, its effects are unlikely to be explained by a single metabolite-target mechanism. We therefore established an experimental analytical framework integrating colonic transcriptomics, fecal microbiota profiling, GI transit assessment, and ELISA-based measurement of motility-related mediators. Colonic transcriptomics was used to examine host intestinal transcriptional responses, whereas fecal microbiota profiling was used to characterize TTC-associated microbial changes. These data were interpreted together with physiological outcomes to explore whether TTC intervention was associated with coordinated changes in fecal microbiota composition, colonic host responses, and 5-HT-associated GI motility regulation.

Therefore, this study aimed to evaluate the effects of TTC on LFD-induced constipation and to identify the associated changes in transcriptional responses, microbiota composition, and GI motility regulation.

## Materials and methods

### Chemicals and reagents

Tiantian Capsule (TTC; batch number: 20180923) was provided by Hebei Yuzhilin Pharmaceutical Co., Ltd., (Shijiazhuang, China). TTC was composed of five source materials: dried leaf juice of *Aloe vera* (L.) Burm.f. [Asphodelaceae; Luhui; AV], dried mature fruit pulp of *C. moschata* Duchesne [Cucurbitaceae; Nangua; CM], dried sclerotium of *W. cocos* (F.A.Wolf) Ryvarden and Gilb. [Fomitopsidaceae; Fuling; PC], dried fruiting bodies of *T. fuciformis* Berk. [Tremellaceae; Yiner; TF], and xylooligosaccharides derived from Maize Cob by enzymolysis. The proportions of these source materials were 25%, 25%, 12.5%, 12.5%, and 25%, respectively. Detailed information on taxonomic validation, raw-material suppliers, and processing procedures is provided in Supplementary Table S1.

### Animals and drug administration

Male Sprague–Dawley rats aged 6–8 weeks and weighing 180–220 g (n = 32) were obtained from Vital River Laboratory Animals Ltd. For gavage administration, the contents of TTC and hemp seed soft capsule (HSSC) were accurately weighed and freshly suspended in 0.3% CMC-Na immediately before use. The doses of TTC and the positive control HSSC were selected based on existing literature (Li et al., 2021). Based on body-surface-area normalization, TTC at 36 mg/kg/day and HSSC at 126 mg/kg/day corresponded to human equivalent doses of approximately 0.35 g/day and 1.22 g/day, respectively, for a 60-kg adult. These doses are within orally attainable ranges and are comparable to the recommended human intake stated in the product information. However, because pharmacokinetic bridging was not performed, these estimates should be interpreted only as dose-relevance estimates rather than direct clinical equivalence.

The LFD-induced constipation model was selected because insufficient dietary fiber intake is closely associated with functional constipation and has been used in previous rodent studies to induce constipation-like phenotypes. Previous studies have shown that low-fiber or fiber-deficient diets can reduce fecal output, fecal weight, and fecal water content, delay GI transit, and alter fecal microbiota composition. Therefore, in the present study, model establishment and treatment effects were evaluated using a multidimensional framework, including fecal parameters, GI transit assessment, fecal microbiota profiling, and intestinal histological evaluation (Kakino et al., 2010; Oshiro et al., 2022; Yi et al., 2022; Li et al., 2024).

The Sprague–Dawley rats were housed individually and acclimatized for 1 week with *ad libitum* access to water and standard chow. After acclimatization, the rats were randomly allocated into four groups using body-weight-balanced randomization, with eight rats in each group. Investigators responsible for outcome assessment were blinded to group allocation where applicable. The specific group assignments, modeling methods, and administration protocols were as follows: (1) control group (NC, n = 8): rats were fed a normal diet and administered 0.3% CMC-Na by gavage at 10 mL/kg/day; (2) model group (LFD, n = 8): rats were fed an LFD and administered 0.3% CMC-Na by gavage at 10 mL/kg/day; (3) TTC group (TTC, n = 8): rats were fed an LFD and administered TTC by gavage at 36 mg/kg/day; and (4) HSSC group (HSSC, n = 8): rats were fed an LFD and administered HSSC by gavage at 126 mg/kg/day. The modeling and administration period lasted 5–6 weeks according to the experimental schedule. At week 6, rats were euthanized after the final assessments.

### Detection of physiological and fecal parameters in rats

In order to accurately evaluate the effect of the drugs on the defecation function of constipated rats, corn cob bedding was used in each experimental cage of the animals, which was changed regularly to exclude the influence of other factors, such as urine, on the results. During the experimental period, the feces of each rat in each group were collected at 10:00 a.m. on three consecutive days of each week to count the fecal parameters (fecal pellet number, fecal weight, and water content) and record the data. The number and weight of feces were the total number and weight of feces collected in each group within 24 h. Three fresh feces were collected from each group to analyze the water content of the feces. The fecal water content was determined by drying the samples in an oven at 100 °C for 24 h. The formula was:

$$water\;content\;of\;feces=\frac{wet\;weight\;of\;feces-dry\;weight\;of\;feces}{wet\;weight\;of\;feces}\times 100\%$$

### Hematoxylin and eosin (H&E) staining

Small intestine and colonic tissues were fixed in tissue fixative for more than 24 h, dehydrated through a graded ethanol series, cleared in xylene, embedded in paraffin, and sectioned at 4 μm thickness. After deparaffinization and rehydration, the sections were stained with hematoxylin for 1–2 min, differentiated, blued, and then counterstained with eosin for 2–3 min. The sections were subsequently dehydrated, cleared, and mounted with neutral resin. Histological images were observed and captured using a fluorescence inverted microscope (Carl Zeiss, Germany). H&E staining reagents were purchased from Wuhan Baiqiandu Biological Technology Co., Ltd., (Wuhan, China).

### Alcian blue and periodic acid schiff (AB&PAS) staining

Colonic tissues were fixed, paraffin-embedded, sectioned, deparaffinized, and rehydrated as described for H&E staining. The sections were stained with Alcian blue solution for 5 min, treated with periodic acid solution for 15 min, and then stained with Schiff reagent for 30 min. After hematoxylin counterstaining, differentiation, and bluing, the sections were dehydrated, cleared, and mounted with neutral resin. Images were observed and captured using a fluorescence inverted microscope (Carl Zeiss, Germany). AB&PAS staining reagents were purchased from Wuhan Baiqiandu Biological Technology Co., Ltd., (Wuhan, China).

### Transcriptomic RNA sequencing of colonic tissues

Five colonic tissue samples from each group including NC, LFD and TTC were selected for transcriptomic RNA sequencing. Total RNA was extracted from colonic tissues, and RNA integrity was assessed using an Agilent 2100 Bioanalyzer (Agilent Technologies, USA). Sequencing libraries were prepared using the NEBNext Ultra II Directional RNA Library Prep Kit for Illumina (New England Biolabs, USA) according to the manufacturer’s instructions. The average insert size of the libraries was approximately 380 bp. Sequencing was performed on the Illumina NovaSeq 6000 platform using a paired-end 150 bp strategy.

Raw reads were filtered using Fastp with the parameters -q 20 -u 30 -l 50 -g to remove adapter-contaminated reads, low-quality reads, and short reads. Read quality was assessed using FastQC with default parameters. Clean reads were aligned to the rat reference genome *Rattus norvegicus* mRatBN7.2 using HISAT2. Gene-level read counts were generated using HTSeq in union mode, and gene expression levels were normalized as fragments per kilobase of transcript per million mapped reads (FPKM).

Differential expression analysis was performed using DESeq v1.42.1. Differentially expressed genes were screened using *P* < 0.05 and |log2FC| > 1 as the thresholds. Gene Set Enrichment Analysis (GSEA) was performed using clusterProfiler v4.11.1 to identify enriched Kyoto Encyclopedia of Genes and Genomes (KEGG) pathways. Multiple-comparison correction for RNA-seq and enrichment analyses is described in the Statistical test section.

### 16S rRNA sequencing of fecal microbiota

Fresh fecal samples were collected from each rat using sterile forceps, immediately placed into sterile microcentrifuge tubes on ice to preserve microbial DNA integrity, and stored at −80 °C until DNA extraction. Total microbial DNA was extracted from fecal samples using the E. Z.N.A.® Soil DNA Kit according to the manufacturer’s protocol. DNA quality was assessed by 1% agarose gel electrophoresis, and DNA concentration and purity were measured using a NanoDrop 2000 spectrophotometer.

The V3–V4 region of the bacterial 16S rRNA gene was amplified by PCR using primers 338F and 806R. PCR products from the same sample were pooled, separated on a 2% agarose gel, and purified using the AxyPrep DNA Gel Extraction Kit. The purified amplicons were quantified using a Quantus™ Fluorometer and sequenced on an Illumina MiSeq platform using a PE300 strategy.

After quality control and sequence optimization, operational taxonomic units (OTUs) were clustered at 97% sequence similarity using UPARSE v7.0.1090. Taxonomic annotation of representative OTU sequences was performed using the RDP Classifier v2.11 against the SILVA database Release 138 with a confidence threshold of 0.7.

Alpha-diversity indices, including Sobs, Shannon, and Simpson indices, were calculated to evaluate fecal microbial richness and diversity. Differences among multiple groups were analyzed using the Kruskal–Wallis rank-sum test, followed by pairwise Wilcoxon rank-sum tests where appropriate. Principal coordinate analysis (PCoA) was performed to evaluate similarities and differences in microbial community composition among samples. Taxonomic composition was analyzed at the phylum and genus levels to compare the relative abundance of microbial taxa among groups. Linear discriminant analysis effect size (LEfSe) was used to identify differentially abundant taxa among groups, with significance assessed using the non-parametric Kruskal–Wallis rank-sum test.

### GI transit function

At the end of administration, three rats from each group were randomly selected for GI transit assessment. At 8:00 a.m., each rat was gavaged with 1 mL saline containing six stainless steel beads (diameter = 1.0 mm). Barium sulfate suspension (15%, 2 mL) was administered by gavage as a contrast agent to outline the GI tract. Rats were anesthetized with isoflurane and subjected to X-ray imaging at 4, 9, and 12 h after bead administration. The anatomical location of each bead was recorded according to predefined GI segments.

The GI transit score was calculated as the sum of the scores assigned to the six beads in each rat. Each bead was scored according to its location as follows: 0, stomach; 1, small intestine; 2, cecum; 3, colon; 4, rectum; and 5, outside the GI tract. Thus, the total GI transit score for each rat ranged from 0 to 30, with higher scores indicating more distal bead progression and faster GI transit. Gastric emptying was calculated as the percentage of beads that had exited the stomach at each time point. X-ray images were evaluated by investigators blinded to group allocation.

### ELISA for GI hormones and neurotransmitters

Serum levels of motilin (MTL), gastrin (GAS), substance P (SP), 5-hydroxytryptamine (5-HT), vasoactive intestinal peptide (VIP), endothelin-1 (ET-1), and somatostatin (SS) were measured using commercial ELISA kits according to the manufacturer’s instructions. All ELISA kits were purchased from Wuhan Xinqidi Biological Technology Co., Ltd., (Wuhan, China). The catalog numbers were as follows: GAS, EIA05702r; MTL, EIA06085r; SP, EIA06357r; 5-HT, EIA05006r; VIP, EIA06521r; ET-1, EIA05635r; and SS, EIA06378r.

### Statistical test

All experimental data were expressed as mean ± standard deviation (SD). For non-omics pharmacological assays involving more than two groups, one-way ANOVA was first performed, followed by Holm-adjusted *post hoc* pairwise t-tests using the pairwise.t.test() function in R 4.3.1. For RNA-seq analysis, differentially expressed genes were screened using the indicated *P*-value and fold-change thresholds, and Benjamini–Hochberg false discovery rate (BH-FDR)-adjusted *P*-values were calculated and reported where applicable. Pathway enrichment analysis and GSEA were performed with BH-based multiple-comparison correction.

For gut microbiota analysis, alpha-diversity indices were analyzed using the Kruskal–Wallis rank-sum test for multi-group comparisons, with FDR correction applied for multiple comparisons. Taxonomic relative abundance between two groups was compared using the Wilcoxon rank-sum test. Spearman’s correlation analysis was used to assess associations among fecal parameters, GI hormone/neurotransmitter levels, and the relative abundance of microbial taxa. Unless otherwise specified, *P* < 0.05 or adjusted *P* < 0.05 was considered statistically significant.

## Results

### Experimental overview

**FIGURE 1:**
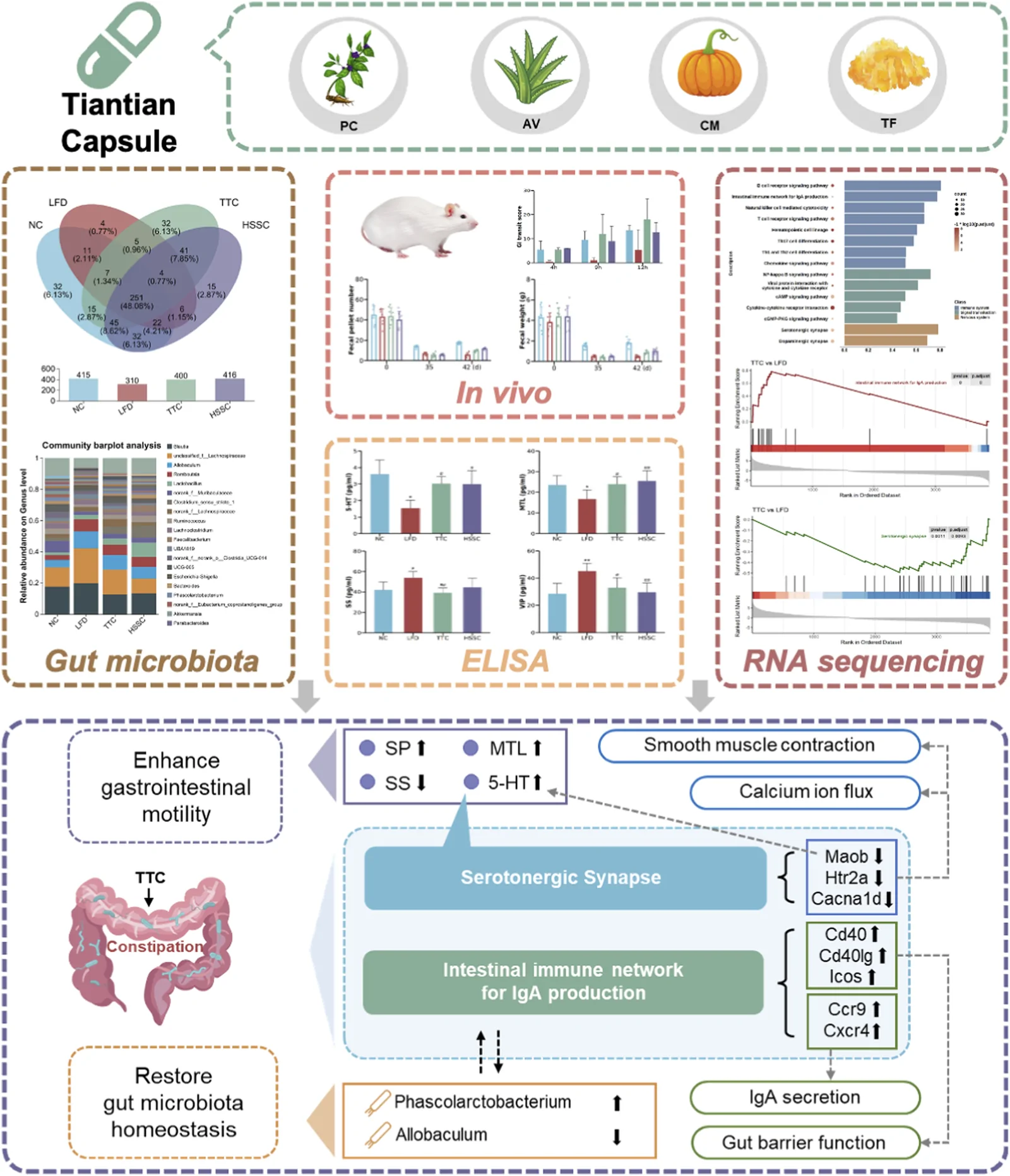
Graphical overview of the experimental design and proposed mechanisms of TTC in LFD-induced constipation.

To evaluate the effects of TTC on LFD-induced constipation, we integrated fecal-parameter assessment, intestinal histological staining, GI transit assessment, ELISA-based measurement of GI hormones/neurotransmitters, colonic RNA sequencing, and fecal microbiota profiling. As shown in Figure 1, these assays were used to assess defecation function, intestinal mucosal and mucus-layer alterations, GI motility, 5-HT-associated neuroendocrine changes, colonic transcriptional responses, and fecal microbiota remodeling. This experimental framework allowed us to examine TTC-associated physiological, host transcriptional, and microbial changes in LFD-induced constipated rats.

### TTC improved defecation function and intestinal mucosal alterations in LFD-induced constipated rats

**FIGURE 2:**
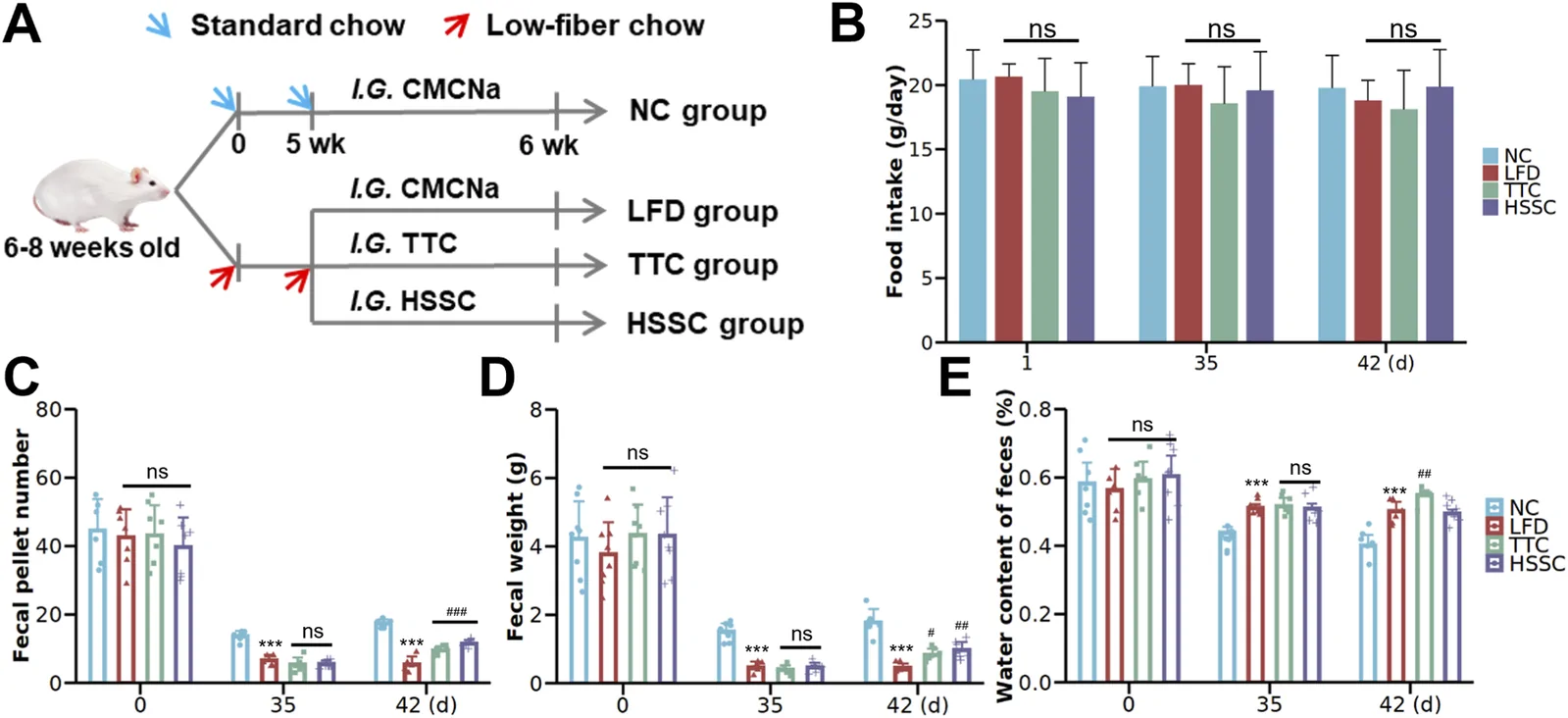
Effect of TTC on defecation function in LFD-induced constipated rats. **(A)** Experiment design and schedule. **(B)** Food intake. **(C)** Fecal pellet number. **(D)** Fecal weight. **(E)** Fecal water content. Data are expressed as mean ± SD. n = 8 rats per group. *p < 0.05, **p < 0.01, ***p < 0.001 vs. the NC group; #p < 0.05, ##p < 0.01, ###p < 0.001 vs. the LFD group; n.s., not significant.

The experimental design and schedule are shown in Figure 2A. During the LFD modeling period, no significant differences in food intake or body weight were observed among the groups (Figure 2B; Supplementary Figure S1), suggesting that the LFD intervention did not induce overt systemic toxicity or malnutrition-related body-weight loss. To validate the LFD-induced constipation model and evaluate the effects of TTC on defecation function, fecal parameters were assessed in all four groups after 1 week of administration. On day 35, rats in the LFD group showed significantly decreased fecal weight and pellet count compared with the NC group, indicating the establishment of a constipation-like phenotype. After 1 week of treatment, TTC significantly increased fecal water content, fecal weight, and pellet count compared with the LFD group. HSSC also significantly increased fecal weight and pellet count (Figures 2C–E). These results indicate that TTC improved defecation function in LFD-induced constipated rats, with an effect direction comparable to that of the positive control HSSC.

**FIGURE 3:**
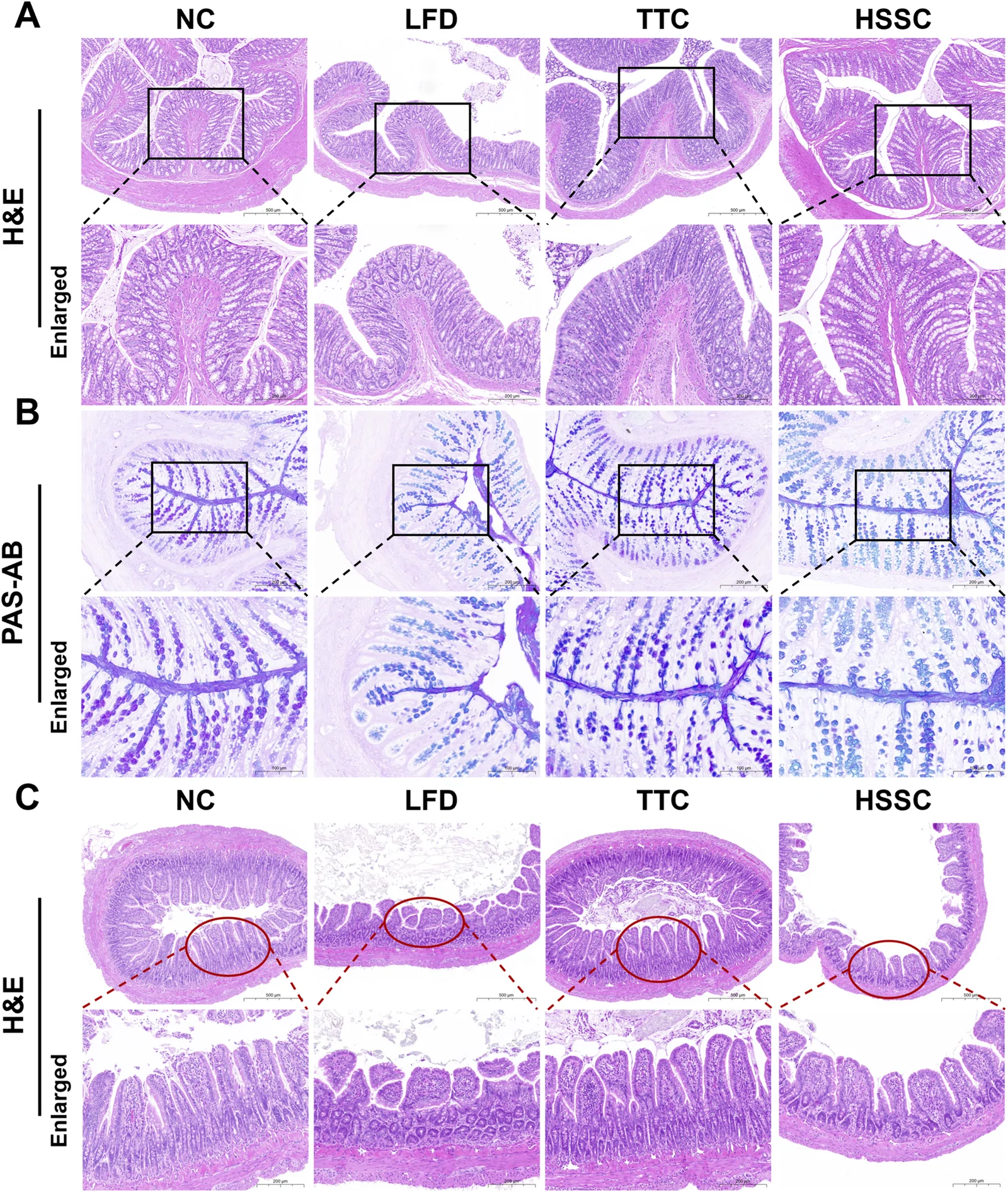
Effect of TTC on histopathology examination of the colon and small intestine in LFD-induced constipated rats. **(A)** Histopathological assay for colon by H&E staining (magnification, ×100, ×200). **(B)** Histopathological assay for colon by AB&PAS staining (magnification, ×100, ×200). **(C)** Histopathological assay for small intestinal by H&E staining (magnification, ×100, ×200).

Histological staining was further used as supportive evidence to assess intestinal mucosal morphology and mucus-related alterations rather than as the primary criterion for model establishment. Colonic H&E staining showed that rats in the NC group exhibited an intact mucosal structure, neatly arranged epithelial cells, and abundant goblet cells, without obvious inflammatory infiltration. In contrast, the LFD group showed reduced goblet cells and mild mucosal alterations. TTC and HSSC treatment increased goblet cell abundance and improved colonic mucosal morphology (Figure 3A). AB&PAS staining further showed that the colonic mucus layer was reduced in the LFD group, whereas TTC and HSSC treatment increased mucus-layer staining (Figure 3B). In addition, H&E staining of small intestinal tissues showed relatively well-organized villi and crypt structures in the NC group, whereas the LFD group displayed decreased goblet cells and impaired villus/crypt morphology. TTC and HSSC treatment improved villus organization and goblet cell abundance compared with the LFD group (Figure 3C). Together, these findings suggest that TTC ameliorated LFD-associated defecation dysfunction and intestinal mucosal/mucus-layer alterations.

### Colonic transcriptomics suggested TTC-associated changes in serotonergic and immune-related pathways

**FIGURE 4:**
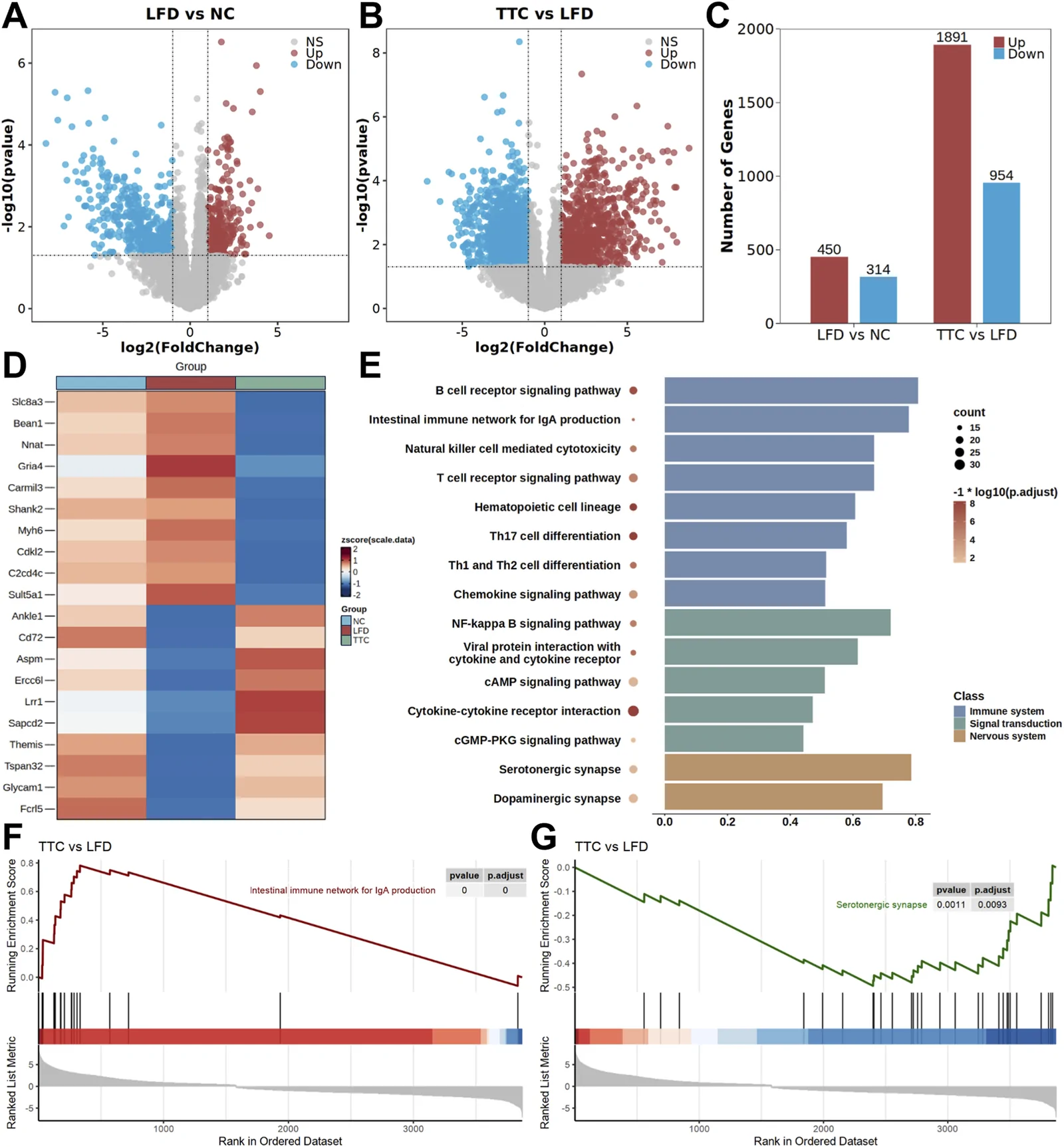
Colonic transcriptomic analysis of TTC intervention in LFD-induced constipation. **(A)** Volcano plot of DEGs between the LFD and NC group. **(B)** Volcano plot of DEGs between the TTC and LFD group. DEGs were screened using *P* < 0.05 and |log2FC| > 1. **(C)** Numbers of upregulated and downregulated DEGs among the NC, LFD, and TTC groups. **(D)** Heatmap of representative DEGs among the NC, LFD, and TTC groups. **(E)** Significantly enriched KEGG pathways identified by GSEA between the TTC and LFD groups. Pathways with adjusted *P* < 0.05 were considered significant. **(F)** GSEA plot of the Intestinal immune network for IgA production pathway. **(G)** GSEA plot of the serotonergic synapse pathway. n = 5 colonic tissue samples per group.

To explore colonic transcriptional changes associated with TTC intervention in LFD-induced constipation, transcriptomic RNA sequencing was performed on colonic tissues from the NC, LFD, and TTC groups. Differential expression analysis identified differentially expressed genes (DEGs) between the LFD and NC groups and between the TTC and LFD groups using the predefined statistical thresholds. Volcano plots of these comparisons are shown in Figures 4A,B. Compared with the NC group, the LFD group showed 450 upregulated and 314 downregulated DEGs. Compared with the LFD group, the TTC group showed 1,891 upregulated and 954 downregulated DEGs (Figure 4C). The expression patterns of representative DEGs across the groups are shown in Figure 4D.

Pathway-level analysis was then performed to identify biological processes potentially associated with LFD-induced constipation and TTC intervention. In the LFD versus NC comparison, enriched pathways were mainly related to immune regulation and signal transduction modules (Supplementary Figure S2). In the TTC versus LFD comparison, enriched pathways included immune-related pathways, particularly the intestinal immune network for IgA production, signal transduction-related pathways such as NF-κB signaling, and nervous system-related pathways, including serotonergic synapse (Figure 4E). These results suggest that TTC intervention was associated with transcriptional changes involving serotonergic signaling and immune-related pathways.

The GSEA enrichment profiles of the serotonergic synapse pathway and the intestinal immune network for IgA production pathway are shown in Figures 4F,G, respectively. In the serotonergic synapse pathway, TTC intervention was associated with decreased expression of genes including *Maob*, *Cacna1d*, *Htr2a*, and *Htr2b*. Notably, TTC intervention was associated with reduced *Maob* expression in the serotonergic synapse pathway. Because MAOB is involved in monoamine metabolism, including serotonin-related metabolism, reduced *Maob* expression may contribute to the maintenance of 5-HT levels by limiting its degradation. Given the established role of 5-HT in regulating gastrointestinal motility (Israelyan et al., 2019), this transcriptional change provides a plausible explanation for the increased serum 5-HT level and improved GI transit observed after TTC treatment. In addition, the decreased expression of *Htr2a* and *Htr2b*, which have been implicated in serotonergic signaling and smooth muscle contraction (Konjevod et al., 2023; Cohen et al., 1985), suggests that TTC may also be associated with altered 5-HT receptor-related motility regulation.

In the Intestinal immune network for IgA production pathway, genes such as *Cd40*, *Cd40lg*, *Icos*, *Ccr9*, and *Cxcr4* were upregulated after TTC intervention. The upregulation of *Ccr9* and *Cxcr4* may promote IgA+ B cells, thereby enhancing IgA secretion (Mora and von Andrian, 2008). Given the role of IgA in maintaining intestinal barrier function and regulating gut microbial colonization (Huus et al., 2021), these changes may be linked to the fecal microbiota remodeling observed after TTC treatment. In addition, the upregulation of *Cd40*, *Cd40lg*, and *Icos* may reflect altered T cell–B cell interactions (Anka Idrissi et al., 2021; Landuyt et al., 2021).

### TTC remodeled fecal microbiota diversity and composition in rats with LFD-induced constipation

**FIGURE 5:**
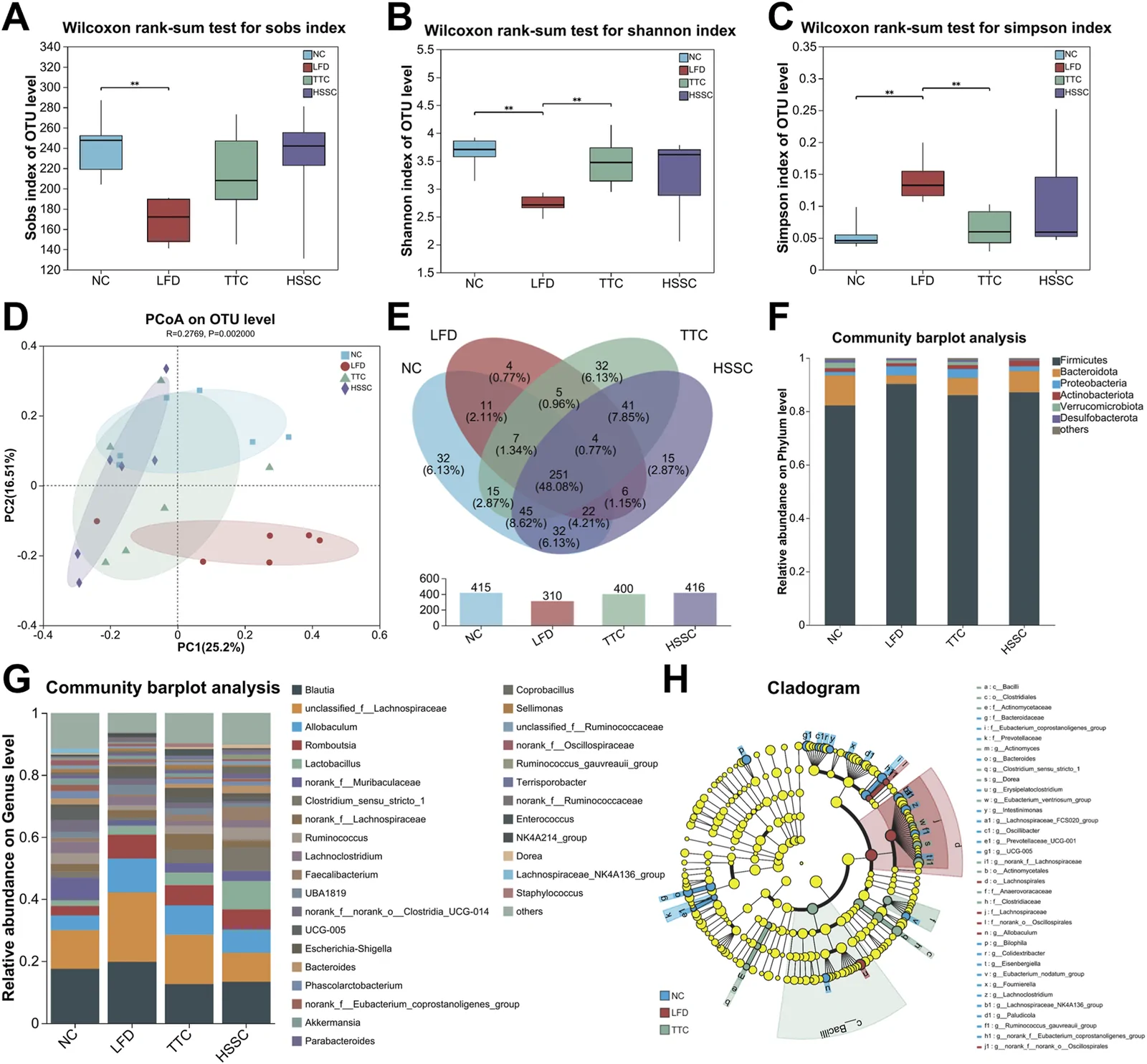
Effects of TTC on fecal microbiota diversity and composition in LFD-induced constipated rats. **(A)** Sobs index at the OTU level. **(B)** Shannon index at the OTU level. **(C)** Simpson index at the OTU level. **(D)** PCoA analysis at the OTU level. **(E)** Venn diagram showing the number of OTUs among different groups. **(F)** Fecal microbiota composition at the phylum level. **(G)** Fecal microbiota composition at the genus level. **(H)** LEfSe analysis showing discriminative taxa among the NC, LFD, and TTC groups. Data are expressed as mean ± SD. *p < 0.05, **p < 0.01, ***p < 0.001 indicating a significant difference.

Because colonic transcriptomic analysis suggested TTC-associated immune-related transcriptional changes, and fecal microbiota is closely linked to intestinal mucosal responses, 16S rRNA sequencing was performed to profile fecal microbiota. After quality control, optimized sequences were clustered into OTUs and assigned taxonomic information. Alpha-diversity analysis showed that microbial richness/diversity was reduced in the LFD group compared with the NC group. Compared with the LFD group, TTC treatment increased the Shannon and Simpson indices, suggesting improved fecal microbial diversity after TTC intervention (Figures 5A–C).

PCoA analysis at the OTU level showed a clear separation between the NC and LFD groups, whereas the TTC and HSSC groups clustered closer to the NC group along the PC2 axis (Figure 5D). Venn diagram analysis showed that the NC, LFD, TTC, and HSSC groups contained 415, 310, 400, and 416 OTUs, respectively, with 251 OTUs shared among all four groups. The NC, LFD, TTC, and HSSC groups contained 32, 4, 32, and 15 unique OTUs, respectively (Figure 5E). These results suggest that LFD feeding altered fecal microbiota composition and that TTC treatment partially shifted the microbial community structure toward that of the NC group.

At the phylum level, the fecal microbiota across the four groups was mainly composed of six phyla, including *Firmicutes*, *Bacteroidetes*, and *Proteobacteria* (Figure 5F). Compared with the NC group, the LFD group showed an increased relative abundance of *Firmicutes* and a decreased relative abundance of *Bacteroidetes*, whereas these changes were partially reversed after TTC and HSSC treatment. At the genus level, the relative abundances of *unclassified_f__Lachnospiraceae*, *Allobaculum*, and *Romboutsia* were increased in the LFD group compared with the NC group and decreased after TTC or HSSC treatment. Conversely, *norank_f_Muribaculaceae*, *Bacteroides*, and *Phascolarctobacterium* were decreased in the LFD group and increased after TTC or HSSC treatment (Figure 5G).

LEfSe analysis was further performed to identify taxa that differed among the NC, LFD, and TTC groups. A total of 36 discriminative bacterial taxa were identified from the phylum to genus levels. *Allobaculum* was enriched in the LFD group, *Bacteroidetes* and *UCG-005* were enriched in the NC group, and *Dorea* was enriched in the TTC group (Figure 5H). Together, these findings indicate that TTC treatment was associated with remodeling of fecal microbiota composition in LFD-induced constipated rats.

### TTC improved GI transit and modulated 5-HT-associated GI hormones/neurotransmitters

**FIGURE 6:**
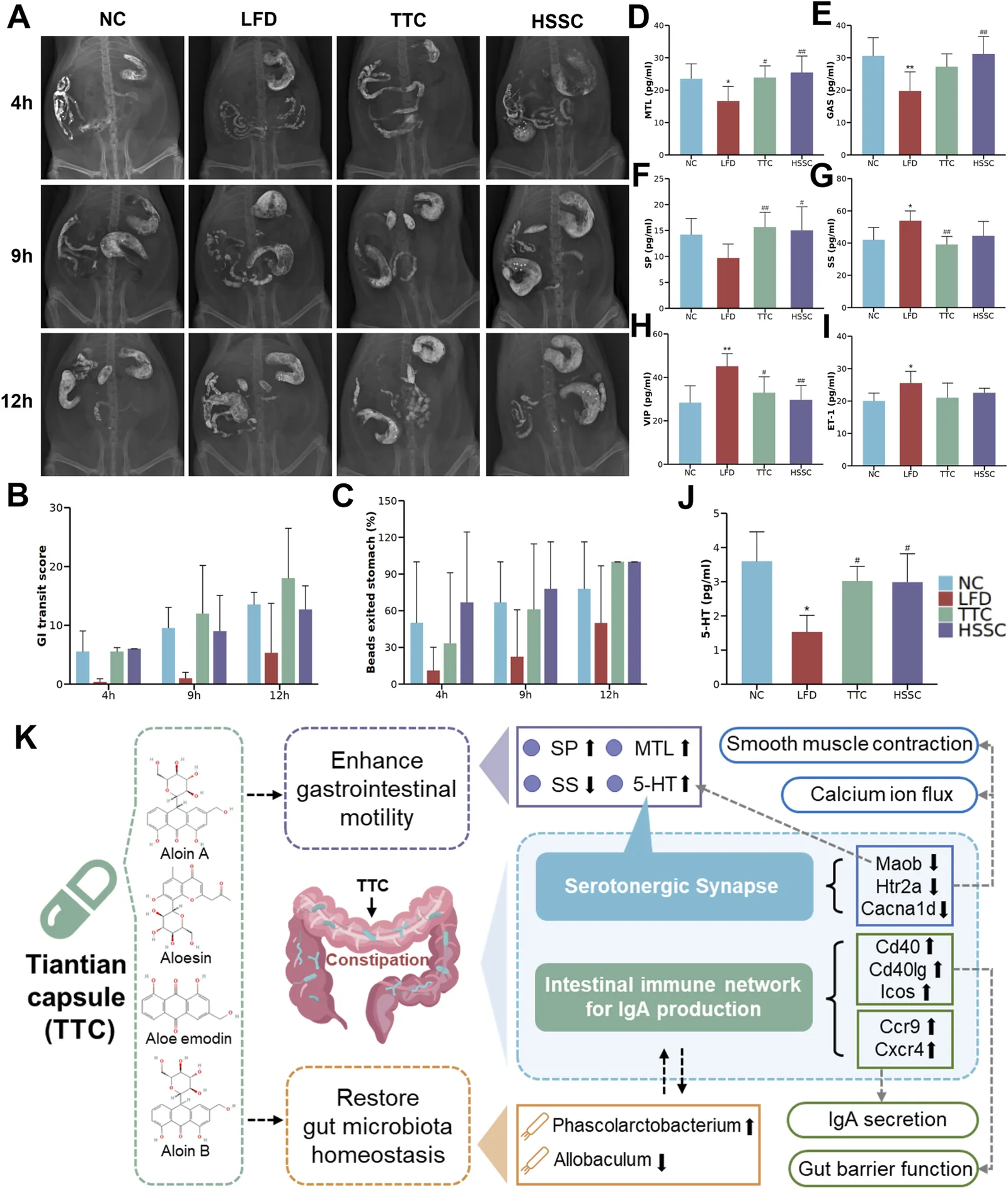
TTC improved GI transit and modulated 5-HT-associated GI hormones/neurotransmitters in LFD-induced constipated rats. **(A)** Representatively selected X-ray images at three time points. **(B)** GI transit score. **(C)** Gastric emptying index. **(D)** MTL levels. **(E)** GAS levels. **(F)** SP levels. **(G)** SS levels. **(H)** VIP levels. **(I)** ET-1 levels. **(J)** 5-HT levels. **(K)** Schematic diagram summarizing the proposed mechanisms underlying the treatment of LFD-induced constipation by TTC. Data are expressed as mean ± SD. *p < 0.05, **p < 0.01, ***p < 0.001 vs. the NC group; #p < 0.05, ##p < 0.01, ###p < 0.001 vs. the LFD group; n.s., not significant.

Based on the transcriptomic findings, we further examined whether TTC intervention was associated with changes in GI motility and motility-related hormones/neurotransmitters, particularly 5-HT. GI transit was assessed using X-ray imaging to track the distribution of six stainless-steel beads within the GI tract at 4, 9, and 12 h after gavage. Representative abdominal X-ray images from each group are shown in Figure 6A.

Compared with the NC group, the LFD group showed significantly reduced GI transit scores at 4, 9, and 12 h after gavage. In contrast, TTC and HSSC treatment increased GI transit scores compared with the LFD group, and significant differences remained evident at 12 h (Figure 6B). These results indicate that TTC improved delayed GI transit in LFD-induced constipated rats. Similarly, the gastric emptying index, calculated as the percentage of beads exiting the stomach, was lower in the LFD group than in the NC group at 4, 9, and 12 h. TTC and HSSC treatment showed an increasing trend in gastric emptying index compared with the LFD group (Figure 6C), suggesting partial improvement of LFD-associated delayed gastric emptying.

To further examine motility-related mediators suggested by transcriptomic analysis, serum levels of GI hormones and neurotransmitters were measured by ELISA. Compared with the NC group, the LFD group showed significantly decreased levels of 5-HT, MTL, GAS, and SP. TTC administration significantly increased the levels of these mediators compared with the LFD group. Conversely, VIP, ET-1, and SS levels were significantly elevated in the LFD group, whereas TTC treatment reduced these levels to varying degrees. In the HSSC group, changes in ET-1 and SS did not reach statistical significance (Figures 6D–J). Together, these findings suggest that TTC improved GI transit in association with modulation of 5-HT and other motility-related GI hormones/neurotransmitters.

To explore potential links between TTC-associated microbiota changes and constipation-related outcomes, Spearman correlation analysis was performed at the genus level (Supplementary Figure S3). The relative abundance of *Allobaculum* increased in the LFD group and decreased after TTC treatment. Correlation analysis showed that *Allobaculum* was negatively correlated with fecal weight, fecal pellet count, and 5-HT levels, and positively correlated with VIP. Conversely, *Phascolarctobacterium* was decreased in the LFD group and increased after TTC treatment. This genus was positively correlated with fecal weight, fecal pellet count, 5-HT, and GAS, and negatively correlated with ET-1. Similarly, *Bacteroides* and *UCG-005* were decreased in the LFD group and increased after TTC treatment. Both genera were positively correlated with fecal parameters and 5-HT levels, while UCG-005 was negatively correlated with VIP and ET-1.

These findings suggest that TTC intervention was associated with coordinated improvements in GI transit, 5-HT-associated neuroendocrine regulation, and fecal microbiota composition (Figure 6K). Rather than establishing direct causality, these results provide experimental evidence supporting a working model in which TTC may alleviate LFD-induced constipation through combined effects on GI motility-related mediators and fecal microbiota remodeling.

## Discussion

This study used an integrated analytical approach combining colonic transcriptomics, fecal microbiota profiling, and ELISA-based measurement of GI hormones/neurotransmitters to evaluate the effects of TTC on LFD-induced constipation. In animal experiments, TTC improved defecation parameters and ameliorated intestinal mucosal and mucus-layer alterations in LFD-induced constipated rats. Colonic transcriptomic analysis further suggested that TTC intervention was associated with pathways related to GI motility and intestinal immune responses. Together with GI transit assessment, ELISA results, and fecal microbiota profiling, these findings support a working model in which TTC may alleviate LFD-induced constipation through coordinated changes in 5-HT-associated GI motility regulation, GI hormone/neurotransmitter balance, and fecal microbiota composition. These results provide experimental evidence supporting the potential application of TTC as a functional food for constipation management, while further mechanistic validation is still required.

Before the intervention experiments, HPLC fingerprinting was performed for TTC quality characterization, and representative marker peaks were identified. By comparing the TTC sample with reference standards, Aloe-derived marker metabolites, including aloin A, aloin B, aloe-emodin, and aloesin, were identified (Supplementary Figure S4). AV, a plant used in medicines and foods, has been widely used in functional foods and cosmetics and has also been used in traditional medicine preparations (Yu et al., 2024). Previous studies have suggested that AV, when combined with other source materials in TTC, may exhibit intestinal protective effects, including anti-inflammatory and immunomodulatory activities. These effects may be related to altered *in vivo* processes after formulation with other source materials (Zhang et al., 2024). Given the established use of AV-derived preparations in constipation management, these Aloe-derived marker metabolites provide useful chemical clues for future studies.

Several animal models have been used for constipation research, including drug-induced models such as loperamide-induced constipation and diet-related models such as low-fiber or fiber-deficient diet-induced constipation. Drug-induced models are useful for evaluating pharmacologically suppressed intestinal motility or secretion, whereas LFD-induced models are more relevant to constipation associated with insufficient dietary fiber intake and are therefore suitable for evaluating food-derived or plant/fungal-source interventions such as TTC (Kakino et al., 2010; Oshiro et al., 2022; Yi et al., 2022; Li et al., 2024). However, the LFD model generally represents a milder functional constipation phenotype, and severe histopathological lesions are not necessarily expected. Therefore, model validation in this study was based primarily on fecal parameters and GI transit, with histology used as supportive evidence.

To explore host intestinal responses associated with TTC intervention, colonic transcriptomic profiling was performed in colonic tissues from the NC, LFD, and TTC groups. Pathway-level analysis of DEGs suggested that TTC intervention was associated with transcriptional changes related to serotonergic synapse and intestinal immune network for IgA production pathways. Among these DEGs, *Maob* was downregulated after TTC intervention. Previous studies have reported that reduced *Maob* expression may be associated with increased 5-HT levels (Konjevod et al., 2023), which is consistent with the increased serum 5-HT observed in this study. In addition, *Cacna1d*, which encodes a voltage-gated Ca^2+^ channel, and *Htr2a*, which encodes a 5-HT2 receptor, are involved in smooth muscle contraction and serotonergic signaling (Liu et al., 2021; Cohen et al., 1985), suggesting a possible link between these transcriptional changes and GI motility regulation. In the intestinal immune network for IgA production pathway, the upregulation of *Cd40*, *Cd40lg*, and *Icos* may reflect altered T cell–B cell interactions (Anka Idrissi et al., 2021; Landuyt et al., 2021). Moreover, the upregulation of *Ccr9* and *Cxcr4*, which have been linked to the localization and function of IgA-producing B cells in the intestinal mucosa, may indicate a transcriptional shift toward IgA-related mucosal immune responses (Mora and von Andrian, 2008; Guo et al., 2021). However, because IgA secretion, immune-cell populations, and target-level protein changes were not directly measured, these findings should be interpreted as exploratory transcriptomic clues rather than confirmed functional immune mechanisms.

The brain-gut axis provides an important bidirectional communication route between the nervous system and the gut, has been implicated in the pathophysiology of constipation. (Jin et al., 2020). Enteroendocrine cells (EECs), specialized epithelial cells located in the GI mucosa, secrete a broad range of bioactive mediators (Cr et al., 2018). These mediators, including GI hormones, neuropeptides, and neurotransmitters, facilitate communication between the central nervous system, the enteric nervous system, and GI effector cells to regulate GI motility and secretion, thereby mediating the interaction between the brain and the gut (Foster and McVey Neufeld, 2013). Motility-related mediators examined in this study included MTL, GAS, SP, VIP, 5-HT, SS, and ET-1. MTL promotes gastric emptying by contracting the gastric body and antrum while relaxing the pylorus (Feighner et al., 1999). GAS enhances GI motility partly by increasing sphincter tone in response to stimuli (Zhang et al., 2014). SP acts as an excitatory neurotransmitter, strengthening smooth muscle contraction and accelerating motility (Hwang et al., 2018). VIP, functioning as both a hormone and inhibitory neurotransmitter, reduces muscle tension and visceral resistance (Van Geldre and Lefebvre, 2004). 5-HT is a key signaling molecule involved in intestinal motility regulation (Sharkey and Wiley, 2016), while SS generally inhibits excitatory signaling and slows GI transit (Ben-Shlomo et al., 2005). Elevated ET-1 has also been associated with impaired colonic motility and delayed transit in constipation-related settings (Duan T. et al., 2023). In the present study, TTC treatment improved GI transit and modulated circulating levels of 5-HT and other motility-related GI hormones/neurotransmitters in LFD-induced constipated rats.

To explore the potential involvement of fecal microbiota in TTC-associated constipation relief, 16S rRNA sequencing was performed to characterize fecal microbial changes after TTC intervention. Spearman correlation analysis was further used to examine associations between key bacterial genera, fecal parameters, and motility-related GI hormones/neurotransmitters. Alpha-diversity analysis showed that TTC treatment significantly increased the Shannon and Simpson indices, suggesting improved fecal microbial diversity in LFD-induced constipated rats. Correlation analysis showed that *Phascolarctobacterium* was positively correlated with SP and 5-HT levels and negatively correlated with ET-1 levels. Its relative abundance was decreased in the LFD group and increased after TTC treatment. In addition, *Bacteroides* and *UCG-005* were positively correlated with 5-HT levels, whereas *Allobaculum* was negatively correlated with 5-HT levels. Previous studies have reported that *Phascolarctobacterium* and *Bacteroides* can produce short-chain fatty acids (SCFAs), which may stimulate EECs and promote colonic 5-HT production (Watanabe et al., 2012; Reigstad et al., 2015; Duan H. et al., 2023). Therefore, TTC-associated fecal microbiota remodeling may be linked to 5-HT-related GI motility regulation, supporting a potential microbiota–5-HT–motility axis. However, because SCFAs and microbiota perturbation were not directly assessed, this axis should be interpreted as an exploratory hypothesis requiring further validation.

This study has several limitations. Although HPLC fingerprinting was performed for TTC quality characterization, the specific metabolites or source materials responsible for the observed effects were not determined, and metabolite-specific target engagement, pharmacokinetics, bioavailability, and tissue exposure were not assessed. The potential contribution of xylooligosaccharides to TTC-associated constipation relief was not functionally evaluated, and downstream microbial fermentation products such as SCFAs were not quantified. In addition, the microbiota findings and integrative correlation analysis remain associative and exploratory because antibiotic treatment, fecal microbiota transplantation, microbial metabolite-rescue experiments, or fully sample-matched perturbation studies were not performed. The relatively small RNA-seq sample size and large number of DEGs may also limit the robustness of gene-level conclusions; therefore, transcriptomic results should be interpreted mainly as exploratory pathway-level evidence. Finally, although transcriptomics and ELISA data suggest the involvement of 5-HT-associated signaling and GI hormone/neurotransmitter changes, direct target-level validation and loss-of-function experiments are still needed. Future studies should include larger cohorts, chemically characterized TTC fractions, targeted molecular validation, SCFA quantification, and microbiota-perturbation experiments.

## Conclusion

In summary, this study showed that TTC ameliorated constipation-related symptoms and improved intestinal histopathological alterations in an LFD-induced constipation model. TTC treatment was associated with improved GI motility and modulation of 5-HT and other GI hormone/neurotransmitter levels. In addition, TTC reshaped fecal microbiota composition and was accompanied by transcriptomic changes related to intestinal immune and signaling pathways. These findings suggest that TTC may improve LFD-induced constipation through coordinated effects on GI motility, neuroendocrine regulation, and microbiota remodeling, supporting its potential as a functional food for constipation management.

## Data Availability

The datasets presented in this study can be found in online repositories. The names of the repository/repositories and accession number(s) are here: NCBI Sequence Read Archive (SRA) under BioProject accession number PRJNA1514853.

## Glossary

TTC: Tiantian capsules
AV: Aloe vera
PC: Poria cocos
CM: Cucurbita moschata
TF: Tremella fuciformis
LFD: low-fiber diet
TCM: traditional Chinese medicine
ELISA: enzyme-linked immunosorbent assay
KEGG: Encyclopedia of Genes and Genomes
GSEA: Gene Set Enrichment Analysis
SPF: specific pathogen-free
CMC-Na: sodium carboxymethyl cellulose
NC: control group
HSSC: Hemp seed soft capsule
GI: gastrointestinal
H&E: Hematoxylin and eosin
AB&PAS: Alcian Blue and Periodic Acid Schiff
MTL: motilin
GAS: gastrin
SP: substance P
5-HT: 5-hydroxytryptamine
VIP: vasoactive intestinal peptide
ET-1: endothelin-1
SS: somatostatin
OTU: operational taxonomic unit
PCoA: principal co-ordinates analysis
LEfSe: linear discriminant analysis Effect Size
SD: standard deviation
DEGs: differentially expressed genes
SCFAs: short-chain fatty acids
EECs: enteroendocrine cells
VGCC: voltage-gated Ca2+ channel
