# Supplementary material for: Tiantian capsule alleviates low-fiber diet-induced constipation with improved 5-HT-associated gastrointestinal motility and gut microbiota remodeling
Source: Front Pharmacol. 2026 Aug 27;17:1755455. doi: 10.3389/fphar.2026.1755455 (PMC13559754; doi:10.3389/fphar.2026.1755455)

Supplementary materials for

Tiantian Capsule alleviates low-fiber diet-induced constipation with improved 5-HT-associated gastrointestinal motility and gut microbiota remodeling

Tingyu Zhang[a, #], Jing Sun[b, c, #], Tiantian Wang[d], Jian Zhang[c], Qingyuan Liu[a], Chayanis Sutcharitchan[a], Lili Li[d], Boyang Wang[a, *], Yinqing Li[d, *], Zhiqi Yin[b]

[a] Bioinformatics Division, Department of Automation, Tsinghua University, Beijing 100084, PR China

[b] Department of TCMs Pharmaceuticals, School of Traditional Chinese Pharmacy, China Pharmaceutical University, Nanjing 211198, Jiangsu, PR China

[c] Affiliated Hospital of Integrated Traditional Chinese and Western Medicine, Nanjing University of Chinese Medicine, Nanjing 210028, Jiangsu, PR China

[d] Hebei Technology Innovation Center of Precise nutrition and health, Hebei Yuzhilin Pharmaceutical Co., Ltd, Shijiazhuang 050000, Hebei, PR China

#: These authors contributed equally.

*: Corresponding author:

Boyang Wang, Institute for TCM-X, Department of Automation, Tsinghua University, Beijing, 100084, China.

E-mail address: wang-by20@mails.tsinghua.edu.cn

Yinqing Li, Hebei Technology Innovation Center of Precise nutrition and health, Hebei Yuzhilin Pharmaceutical Co., Ltd, Shijiazhuang 050000, PR China.

E-mail address: liagh@126.com

Supplementary Methods

Materials, taxonomic validation, and preparation of TTC

The taxonomic names of all plant and fungal materials were validated using internationally recognized taxonomic databases, including Plants of the World Online for plant species and MycoBank/GBIF/Species Fungorum Plus for fungal species. The raw materials were provided by qualified suppliers as follows: Aloe leaves were obtained from Yunnan Evergreen Biological Co., Ltd.; Poria sclerotia from Anhui Helin TCM Decoction Pieces Technology Co., Ltd.; mature pumpkin pulp from Chaoyang Dadi Agricultural Products Processing Co., Ltd.; Tremella fruiting bodies from Zhejiang Jiangyuan Mushroom Co., Ltd.; and xylooligosaccharides from Shandong Longlive Bio-technology Co., Ltd (Table. S1).

According to the manufacturer's production records, mature Aloe leaves were washed with water, cut open, and processed after removal of the gel; the leaf juice was then filtered, concentrated, and dried. Poria was soaked, washed, moistened, slightly steamed, peeled, sliced into blocks or thick slices, and sun-dried. Fresh pumpkin was selected, washed, cut, dried, and pulverized when necessary. Fresh Tremella fruiting bodies were cut, rinsed, soaked, sun-dried, and oven-dried. Xylooligosaccharides were prepared from maize cobs and/or maize stalks by steam explosion or high-pressure cooking, followed by enzymatic hydrolysis and purification. For TTC production, the four solid raw materials, namely pumpkin, Poria, Aloe, and Tremella, were ultra-micronized to more than 300 mesh, uniformly mixed with xylooligosaccharides, filled into capsules, packaged, and sterilized by irradiation.

HPLC fingerprint analysis of TTC

HPLC fingerprint analysis was performed to characterize the chemical profile of TTC. For HPLC sample preparation, approximately 0.2 g of TTC contents was accurately weighed and placed in a stoppered conical flask. Methanol (25 mL) was added, and the sample was weighed, sealed, and ultrasonicated for 20 min. After cooling to room temperature, the sample was reweighed, and the lost weight was replenished with methanol. The solution was mixed thoroughly and filtered, and the subsequent filtrate was used for HPLC fingerprint analysis.

Chromatographic separation was conducted on a Waters Symmetry C18 column (4.6 × 250 mm, 5 μm) using an LC-16 HPLC system. Acetonitrile and water were used as mobile phases under gradient elution. The flow rate was 0.5 mL/min, the detection wavelength was 242 nm, the column

temperature was 40°C, and the injection volume was 10 μL. The reference standards including aloin A (Lot: MUST-22112311), aloin B (Lot: DSTDL009701), aloesin (Lot: MUST-23032403), and aloe emodin (Lot: 110795-202011) were purchased from Chengdu Must Bio-Technology, Chengdu Dest Bio-Technology, and the National Institutes for Food and Drug Control (China), respectively.

Characteristic peaks were identified by comparison with reference standards, including aloin A, aloin B, aloesin, and aloe emodin. Method validation included precision, stability, and repeatability tests using aloin A as the reference peak. Ten batches of TTC samples were analyzed using the Similarity Evaluation System for Chromatographic Fingerprint of Traditional Chinese Medicine (2012 edition), and 11 common peaks were identified with similarity values greater than 0.99, supporting the reproducibility of the TTC fingerprint.

Data collection and network analysis

The chemical metabolite information contained in TTC was obtained from the HERB database (Fang et al., 2021), and the structural data for the 207 unique metabolites in TTC were sourced from PubChem. DrugCIPHER, a computational framework designed to predict genome-wide metabolite-target associations based on pharmacotherapeutic and metabolite structure similarities, was used for target prediction (Zhao and Li, 2010). The top 100 predicted targets, ranked by their similarity scores, were defined as the target profile for each metabolite. A statistical modeling hypothesis, proposed in earlier studies, suggests that targets with higher occurrence frequencies across metabolite-target profiles in TCM prescriptions or herbal medicines are more likely to play significant roles in therapeutic interventions (Liang et al., 2014). Using this hypothesis, the holistic targets of the TTC formula and its constituent botanical drugs were identified, with intersections among them illustrated using upset plots.

In addition, Encyclopedia of Genes and Genomes (KEGG) enrichment analyses were conducted on holistic targets of TTC and each individual botanical drug. By considering the pathological mechanisms of constipation, significantly enriched KEGG pathways (adjust P value < 0.05) were identified and categorized into four key modules based on KEGG pathway classification. Using the results from the network target analysis, a multilevel biological network was constructed to illustrate the potential mechanisms of TTC intervention in constipation.

Supplementary Results

HPLC fingerprint characterization of TTC

The HPLC fingerprint method for TTC showed acceptable precision, stability, and repeatability, supporting its use for chemical characterization. As shown in the direct overlay chromatograms (Fig. S4A), 11 common peaks were identified using aloin A as the reference peak across 10 batches of TTC samples. Similarity analysis showed that all batch similarity values were greater than 0.99, indicating good batch-to-batch consistency of the TTC chemical profile (Fig. S4B). By comparison with reference standards, peak 1, peak 5, peak 7, and peak 11 were assigned to aloesin, aloin B, aloin A, and aloe emodin, respectively (Fig. S4C). These Aloe-derived metabolites were used as characteristic quality-control markers for TTC chemical characterization.

Exploratory network analysis of TTC in the treatment of constipation

The metabolites of the four botanical drugs in TTC were gathered from the HERB database. AV contained the largest number of metabolites, comprising 107 metabolites, which accounted for 46.83% of all TTC metabolites. In contrast, CM and TF contained the fewest metabolites, collectively contributing to less than 10% of the total TTC metabolites (Fig. S5A). The target profiles of TTC metabolites at the whole gene level were predicted using the DrugCIPHER algorithm. Subsequently, the holistic targets for TTC and each botanical drug were calculated separately based on the predicted metabolite-target profiles. The intersections of the holistic targets across the different botanical drugs are illustrated in Fig. S5B. The highest number of holistic targets was specific to TF, followed by AV and PC, with 112 targets shared between AV and PC.

KEGG enrichment analysis of the holistic targets of TTC revealed that TTC's predicted targets were enriched in 17 significantly enriched key pathways, which are associated with four main modules: the immune system, signal transduction, the endocrine/digestive system, and the nervous system (Fig. S5C). Previous studies have highlighted a close association between constipation and both the immune system and signal transduction pathways (Dimidi et al., 2017). Pathways related to the endocrine system, such as Bile secretion, have been shown to be closely linked to GI hormone secretion, suggesting that TTC may alleviate constipation by modulating GI hormone secretion (Miedzybrodzka et al., 2021). The significant enrichment of neurotransmitter-related pathways, including those involving dopamine and 5-HT in the nervous system, further suggests that the mechanism of TTC's action in relieving constipation may be related to the regulation of

neurotransmitter secretion.

We separately enriched the holistic targets of the four botanical drugs and found that targets associated with AV and PC were enriched in all four key modules. While targets associated with CM were enriched in the immune-related pathways, and TF's predicted targets were notably enriched in the signal transduction and nervous system modules (Fig. S5D). Finally, we constructed a multilevel biological network by integrating the predicted key modules and pathway information (Fig. S5E).

References

Dimidi, E., Christodoulides, S., Scott, S. M., and Whelan, K. (2017). Mechanisms of Action of Probiotics and the Gastrointestinal Microbiota on Gut Motility and Constipation. Adv Nutr 8, 484–494. doi: 10.3945/an.116.014407

Fang, S.-S., Dong, L., Liu, L., Guo, J., Zhao, L., Zhang, J., et al. (2021). HERB: a high-throughput experiment- and reference-guided database of traditional Chinese medicine. Nucleic Acids Res 49, D1197–D1206. doi: 10.1093/nar/gkaa1063

Liang, X., Li, H., and Li, S. (2014). A novel network pharmacology approach to analyse traditional herbal formulae: the Liu-Wei-Di-Huang pill as a case study. Mol Biosyst 10, 1014–1022. doi: 10.1039/c3mb70507b

Miedzybrodzka, E. L., Foreman, R. E., Lu, V. B., George, A. L., Smith, C. A., Larraufie, P., et al. (2021). Stimulation of motilin secretion by bile, free fatty acids, and acidification in human duodenal organoids. Mol Metab 54, 101356. doi: 10.1016/j.molmet.2021.101356

Zhao, S., and Li, S. (2010). Network-based relating pharmacological and genomic spaces for drug target identification. PLoS One 5, e11764. doi: 10.1371/journal.pone.0011764

Table. S1. Source materials and suppliers of TTC.

Formulation material	Proportion	Family	Source material	Supplier
Aloe vera (L.) Burm.f.	25%	Asphodelaceae	Leaf juice, dried	Yunnan Evergreen Biological Co., Ltd.
Cucurbita moschata Duchesne	25%	Cucurbitaceae	Mature fruit pulp, dried	Chaoyang Dadi Agricultural Products Processing Co., Ltd.
Wolfiporia cocos (F.A.Wolf) Ryvarden & Gilb.	12.5%	Fomitopsidaceae	Sclerotium, dried	Anhui Helin Traditional Chinese Medicine Decoction Pieces Technology Co., Ltd.
Tremella fuciformis Berk.	12.5%	Tremellaceae	Fruiting bodies, dried	Zhejiang Jiangyuan Mushroom Co., Ltd.
Xylooligosaccharides derived from *Zea mays* L.	25%	Poaceae	Cobs and/or stalks, enzymatic hydrolysis	Shandong Longlive Bio-technology Co., Ltd.

Fig. S1. Line graph of changes in body weight of different groups of rats during feeding.

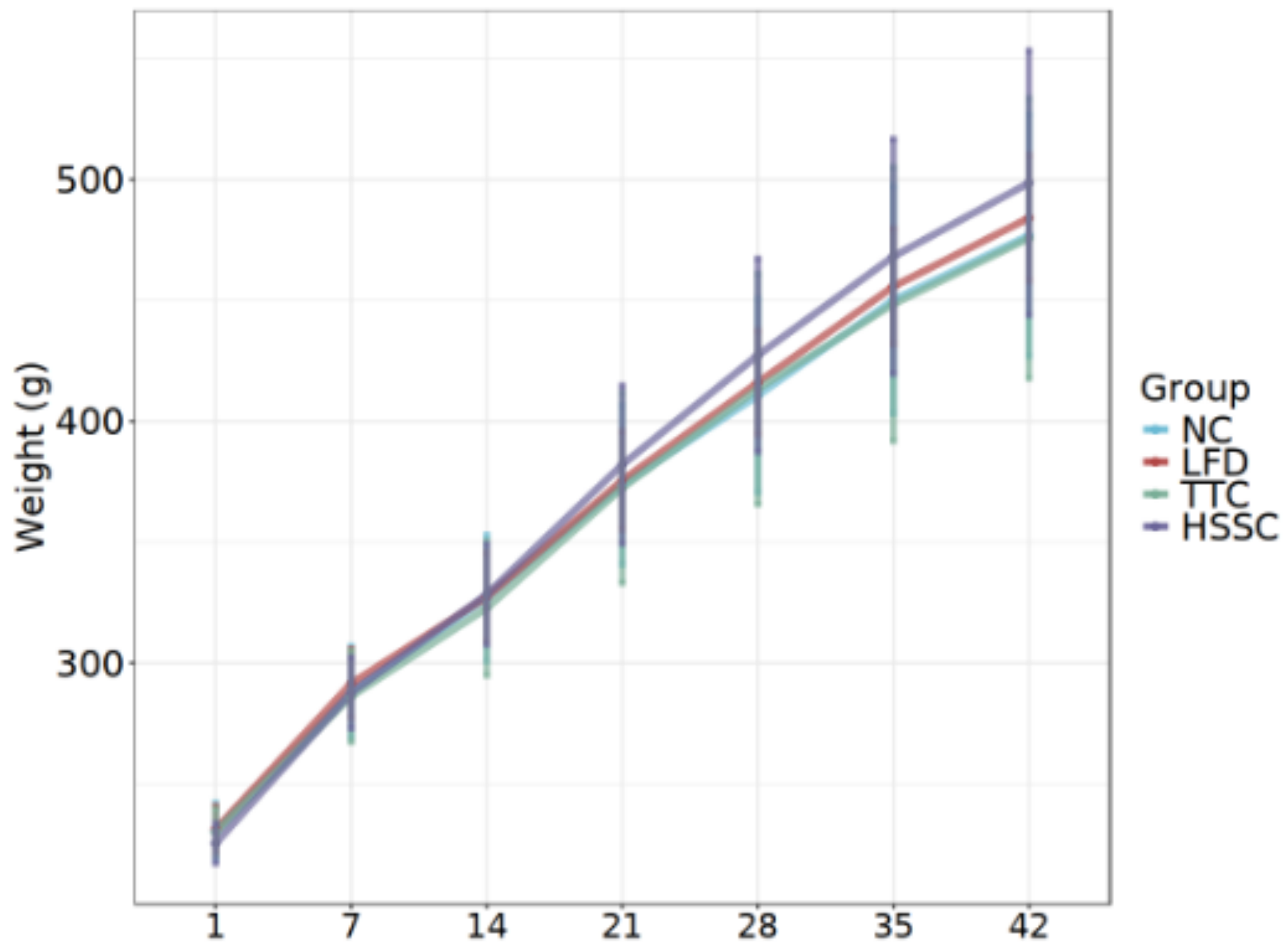

Fig. S2. The enrichment results for the comparison between the LFD and NC groups

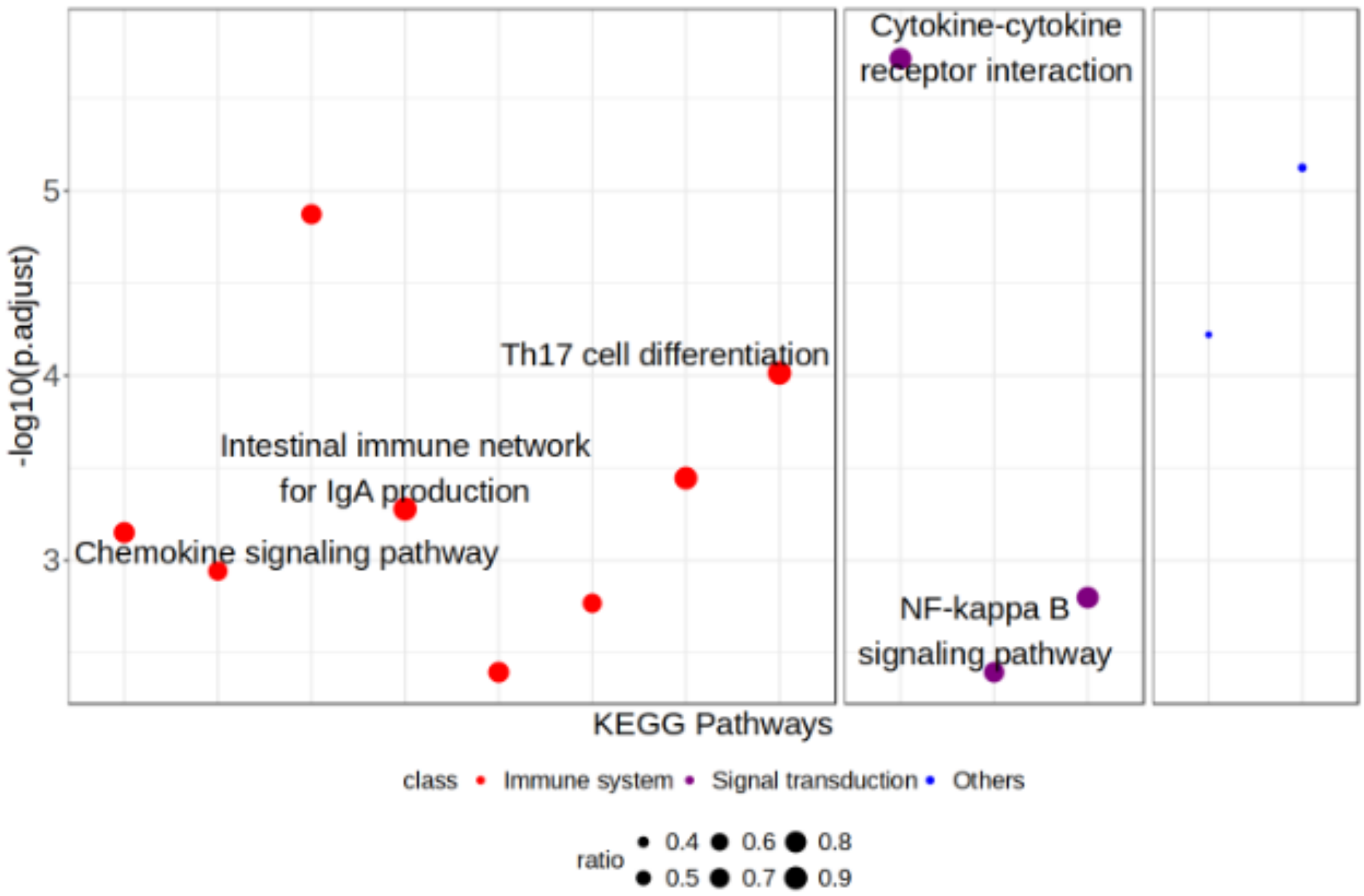

Fig. S3. Spearman correlation analysis. (A) Spearman correlation heatmap between constipation index and genus level. (B) Relative abundance of *Allobaculum*. (C) Relative abundance of *Phascolarctobacterium*. (D) Relative abundance of *Bacteroides*. (E) Relative abundance of *UCG-005*. Data are presented as mean ± SD. *p<0.05, **p<0.01 vs. the NC group; #p<0.05 vs. the LFD group.

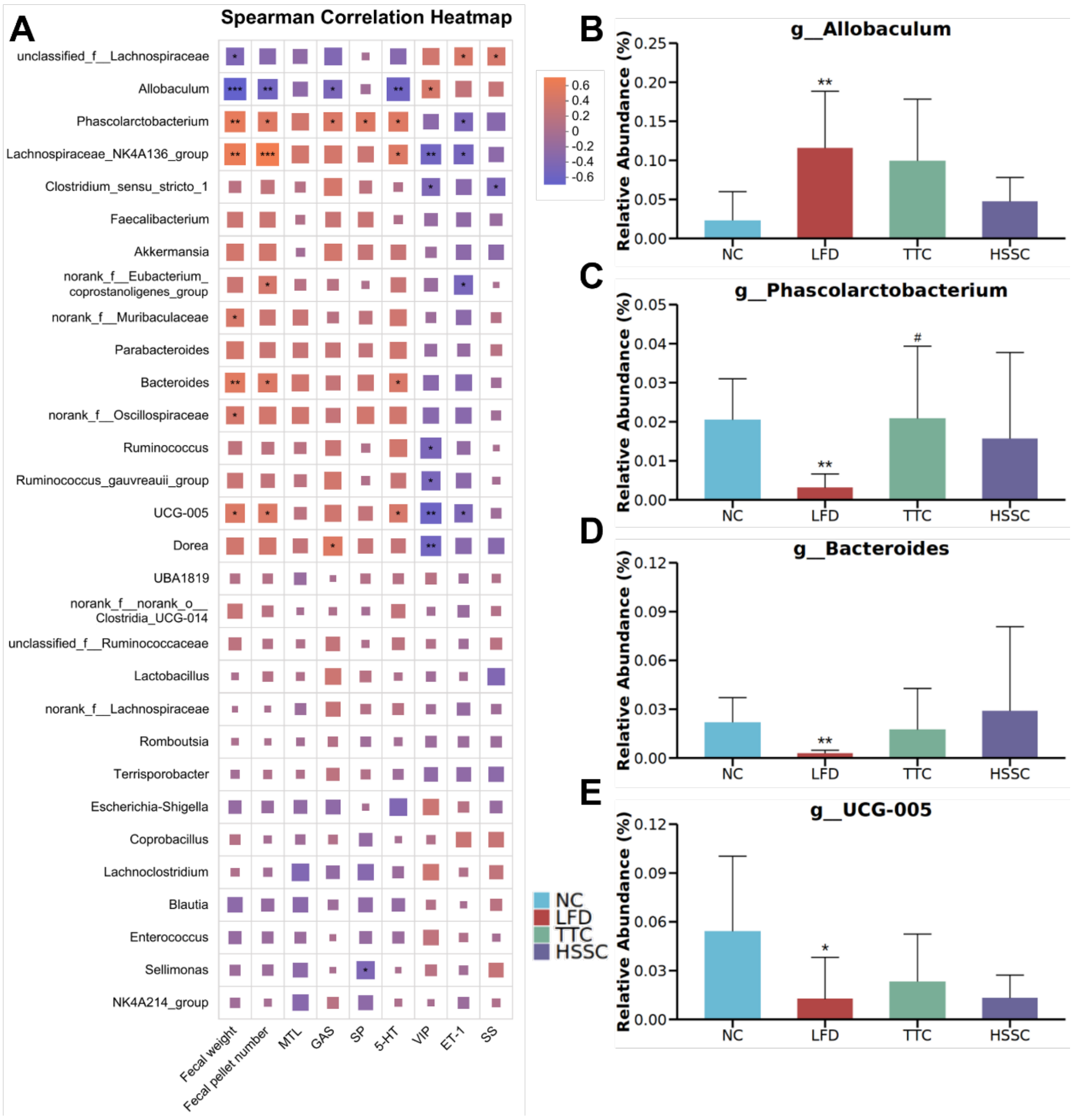

Fig. S4. HPLC fingerprint characterization of TTC. (A) Representative HPLC chromatogram of TTC. (B) HPLC fingerprints of 10 batches of TTC and the reference fingerprint. Eleven common peaks were identified using aloin A as the reference peak, and the chromatographic profiles showed high similarity among batches. (C) Assignment of common peaks in the TTC HPLC fingerprint by comparison with reference standards. Peak 1, peak 5, peak 7, and peak 11 were identified as aloesin, aloin B, aloin A, and aloe emodin, respectively.

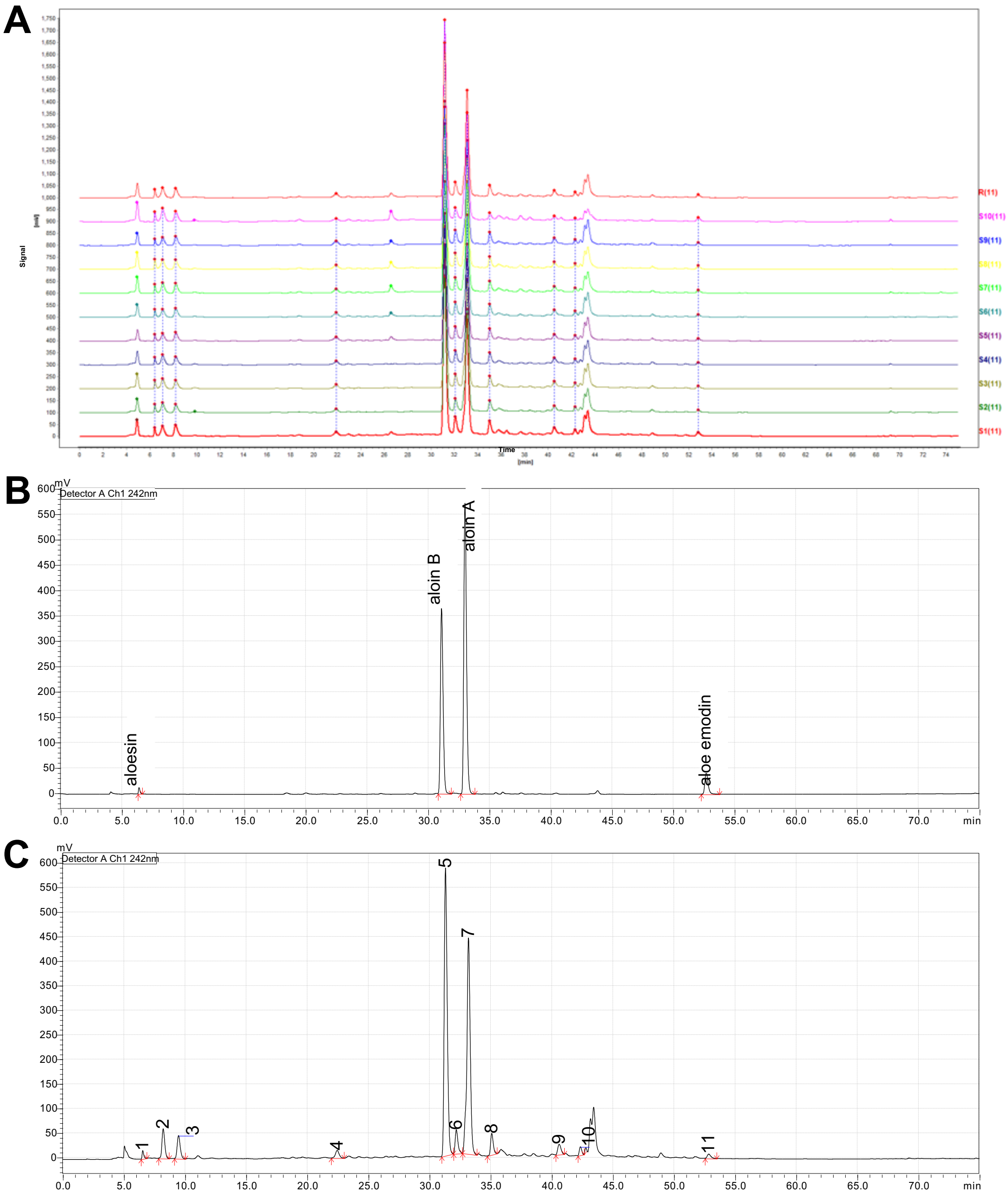

Fig. S5. Exploratory network analysis of TTC in the treatment of constipation. (A) Proportion of metabolites in the four botanical drugs. (B) Upset plot showing the intersections among the holistic targets of the four botanical drugs. (C) Enrichment analysis of the holistic targets of TTC. (D) Enrichment analysis of the holistic targets of the four botanical drugs. (E) Multilevel biological network illustrating the potential mechanisms of TTC in the treatment of constipation.

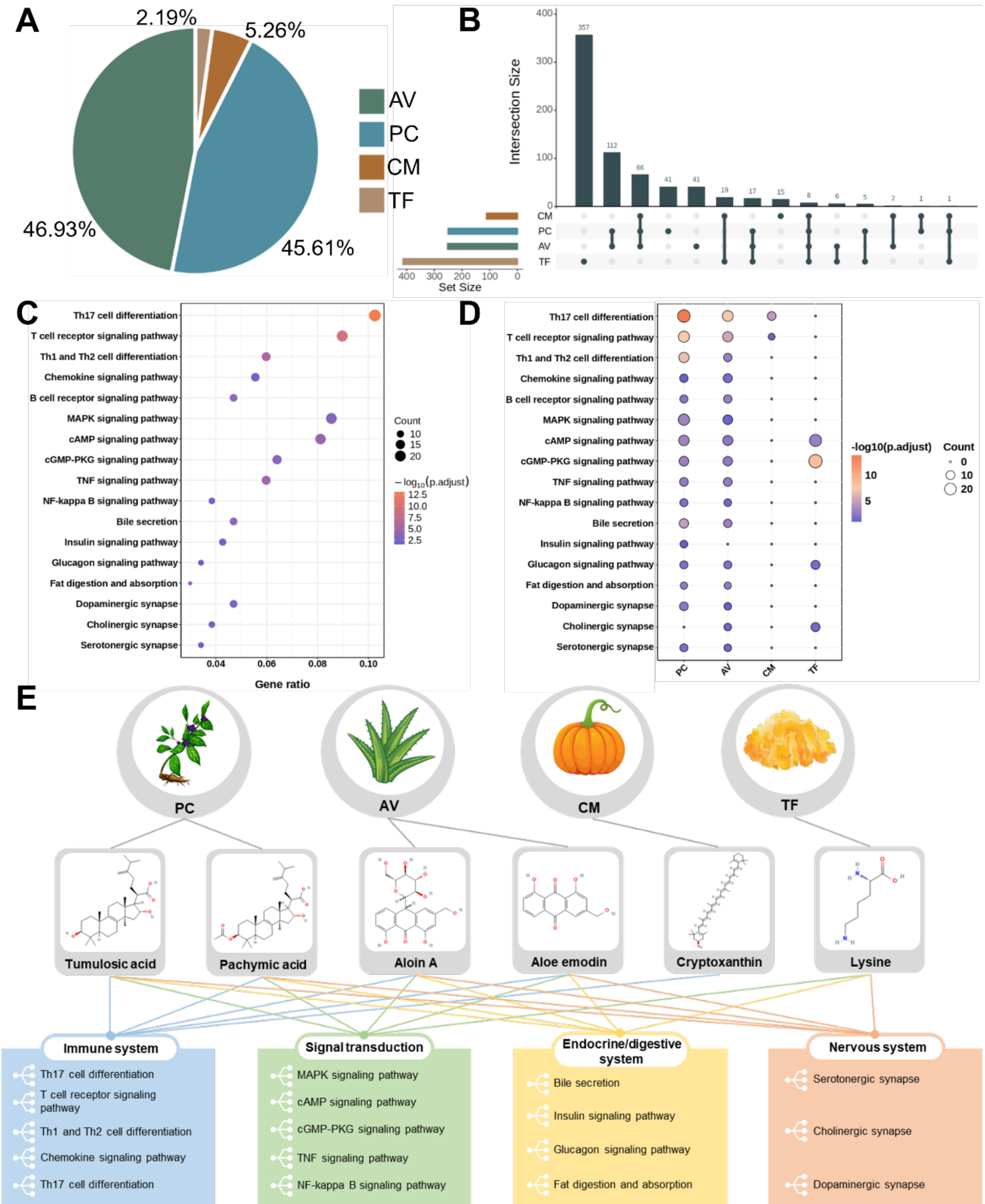

Supplement: Supplementary file 1 [file DataSheet1.PDF]
